# Unraveling the puzzle of phloem parenchyma transfer cell wall ingrowth

**DOI:** 10.1093/jxb/eraa311

**Published:** 2020-07-22

**Authors:** Tyler J McCubbin, David M Braun

**Affiliations:** Divisions of Plant and Biological Sciences, Interdisciplinary Plant Group, and Missouri Maize Center, University of Missouri, Columbia, MO, USA

**Keywords:** Cell wall ingrowths, phloem loading, sucrose, transfer cells

## Abstract

This article comments on:

**Wei X, Nguyen ST, Collings DA, McCurdy DW**. 2020. Sucrose regulates wall ingrowth deposition in phloem parenchyma transfer cells in Arabidopsis via affecting phloem loading activity. Journal of Experimental Botany **71**, 4690–4702.

**For over 100 years, transfer cells have puzzled researchers. Many hypotheses have been proposed to explain the jigsaw-like cell wall ingrowths that characterize the transfer cells found in nearly every angiosperm. Wei *et al.* (2020) investigate the formation and implications of the hallmark cell wall ingrowths of Arabidopsis phloem parenchyma transfer cells (PPTCs) via an elegant combination of genetics and manipulating sucrose availability. In doing so, they shed new light on the role of sugar signaling and carbohydrate export in the development and function of this unique cell type.**

The efflux of photosynthate from leaves and long-distance transport from source to sink tissues is a complex process necessitating specialized cells with diverse functions (Julius *et al.*, 2017). From the time that Münch first proposed the bulk-flow hypothesis (Münch, 1930), the idea of osmotically driven transport of assimilated carbon from the photosynthetic source tissue to distal sink tissues has proven to be a robust, although not fully understood, model (Knoblauch *et al.*, 2016; Knoblauch and Peters, 2017). Even before the advent of modern molecular biology tools, pioneering botanists such as Esau (Esau, 1967; Esau *et al.*, 1953), Evert (Evert *et al.*, 1978), and Fischer (Fischer, 1884) used electron and light microscopy to perform detailed studies of the cellular physiology of the vasculature, which has informed our understanding of the structure and function of cells involved with phloem loading and transport.

## Transfer cells: an early curiosity and earlier adaptation

Broadly, transfer cells are *trans*-differentiated cells that exchange solutes at the interface of an apoplastic–symplastic barrier and are typically characterized by cell wall ‘ingrowths’, or jigsaw-puzzle-like protrusions of cell wall material (Offler *et al.*, 2003). These specialized cells are found in many tissues and organs, from leaves to seeds, to floral nectaries; their ubiquitous presence in nearly every angiosperm and evolutionary conservation to bryophytes suggests they were an early adaptation of land plants (Offler *et al.*, 2003; Pate and Gunning, 1972).

The term ‘transfer cell’ was first used by Fischer in 1884 (Fischer, 1884). Called ‘Übergangszellen’ in his native German, he referred to the dark-staining cells he observed in the minor veins of *Cucurbita.* In what Esau later called ‘the most comprehensive study of minor veins in the past century’ (Esau, 1967), Fischer noted the larger size and greater concentration of organelles in the cytosol of transfer cells in the phloem parenchyma of leaf minor veins, and concluded that the cells were likely to be responsible for transferring photosynthate from the mesophyll to conducting tissues. Nearly 140 years later, we are still trying to unravel the puzzle of exactly how this works. The new research by Wei and colleagues provides many new insights (Wei *et al.*, 2020).

## Curious wall ingrowths

In 1968, Gunning, Pate, and Briarty offered a novel theory of photoassimilate movement centered around the function of the PPTC (Gunning *et al.*, 1968). In their microscopy-heavy tour de force, the authors investigated the peculiar cell wall protrusions of the PPTCs adjacent to the sieve element–companion cell (SE–CC) complex and speculated that the reticulate ingrowths provided a greater surface area to volume ratio than a typical cell, and thus plasmodesmatal density, thereby enhancing solute uptake (see Box 1). The authors estimated a 10-fold increase in this ratio compared with a perfectly smooth cell wall (Gunning *et al.*, 1968), and thus concluded that the PPTC serves as a ‘collection apparatus’. However, slim experimental evidence has been produced to support the hypothesis that the increased surface area enhances solute transport by the PPTC. Notably, however, Wimmers and Turgeon found that the appearance of cell wall ingrowths in minor vein PPTCs positively correlated with light-induced increases in phloem loading and transport rates (Wimmers and Turgeon, 1991), but the mechanisms governing the formation of these unique cell wall ingrowths remained undefined.

## How and why do cell wall ingrowths form?

The processes targeting cell wall ingrowth formation to specific polar domains in the PPTC are unknown. Early research suggested a localized disorganization of the cortical cytoskeleton as a means by which cell wall ingrowths might be deposited in a targeted fashion (Gunning and Pate, 1969). In previous research, McCurdy’s group undertook a detailed study of epidermal transfer cells in *Vicia faba* cotyledons, confirming that reorganization of the microtubule cytoskeleton is associated with cell wall ingrowth deposition. Additionally, they noted that these observations were only made in transfer cells for which cell polarity was already established (Bulbert *et al.*, 1998). The authors concluded that the unique polar organization of microtubules is likely to be required for cell wall ingrowth formation, but the role played by microtubules in the process is not clear.

Additional work aimed at these questions utilized a *V. faba* epidermal cell culture system to profile transcriptional changes associated with *trans*-differentiation to transfer cells (Dibley *et al.*, 2009). The transcriptional changes associated with nascent transfer cell identity implicated auxin and ethylene signaling as fundamental drivers of the *trans*-differentiation process, and therefore cell wall ingrowth deposition (Dibley *et al.*, 2009). These results were confirmed by specific manipulation of ethylene signaling and the finding that blocking ethylene signaling arrested in-progress cell wall ingrowth formation (Zhou *et al.*, 2010). These studies suggest that, at least in an *in vitro* culture system, transfer cell *trans*-differentiation and cell wall ingrowth deposition are conditioned by a cell type-specific burst of ethylene and an up-regulation of particular *ETHYLENE RESPONSE FACTOR* transcription factors (Zhou *et al.*, 2010).

In the present work, Wei and colleagues use a variety of methods to dissect the regulation of PPTC wall ingrowth formation (Wei *et al.*, 2020). Using genetic resources and creative manipulation of growth conditions and media, the authors found that endogenous and exogenous sucrose availability had opposite effects on the development of cell wall ingrowths of Arabidopsis PPTCs. While increasing endogenous sucrose through high light treatment increased cell wall ingrowth, disruption of endogenously produced sucrose through shading or in the *chlorina-1* (Chl *b*-deficient) mutant reduced PPTC wall ingrowth. Contrastingly, exogenous sucrose—but not glucose, fructose, or mannitol-induced osmotic effects—repressed PPTC wall ingrowths in source leaves. Interestingly, the regulation of cell wall ingrowths was found to be independent of hexokinase and trehalose-6-phosphate signaling pathways, suggesting that it is sucrose that specifically acts to control cell wall ingrowth formation in PPTCs of source tissue minor veins. Furthermore, the authors demonstrate that phloem loading activity was strongly associated with cell wall ingrowth deposition, and disruption of phloem loading through physical (e.g. shading) or genetic means reduced PPTC wall ingrowth. Accordingly, the *sweet11; sweet12* double mutant, which has impaired sucrose export and increased sucrose content in the leaves (Chen *et al.*, 2012), exhibited reduced PPTC wall ingrowth, leading the authors to conclude that the formation is stimulated by the activity of phloem loading. The relationship between the sucrose accumulation requirement described by Wei *et al.* (2020) and ethylene signaling processes is not clear, and cultured *Vicia* cotyledon epidermal cells may be subject to different regulatory schema from those of the Arabidopsis minor vein PPTCs.

To date, *GIGANTEA* may be the most interesting player identified as regulating cell wall ingrowth deposition in the PPTC (Edwards *et al.*, 2010). Although well characterized as a circadian-regulated gene involved in promoting flowering in response to photoperiod (Fowler *et al.*, 1999), phytochrome signaling (Huq *et al.*, 2000), and the breaking of seed dormancy (Penfield and Hall, 2009), its role in promoting cell wall ingrowth formation in PPTCs remains enigmatic.

## New questions arise

Altogether, the research by Wei *et al*. presents a major advance in our understanding of PPTC function and development. While the authors clearly demonstrate that the activity of phloem loading stimulates cell wall ingrowth formation, which is congruent with previous hypotheses about increased plasma membrane surface area facilitating greater solute transport, an important next step will be to demonstrate whether SWEET proteins preferentially localize to the cell wall ingrowths to facilitate enhanced sucrose efflux to the apoplast adjacent to the SE–CC. In addition to the specific functional implications of cell wall ingrowth in PPTCs, the identification of endogenous sucrose as a signal for their formation begets further important questions. What sensing mechanism(s) perceives sucrose accumulation in PPTCs such that they are the only cell type in veins to induce cell wall ingrowth formation, and what signal transduction components act downstream to initiate the polar deposition of cell wall ingrowth? Additionally, how PPTC wall ingrowths are targeted to specific domains in the cell wall is still unknown. While the new findings by Wei *et al*. demonstrate that sucrose is a key signal required for cell wall ingrowth formation, the specific mechanisms by which the ingrowths are deposited remain to be elucidated.

It took 80 years for Gunning, Esau, and cohorts to confirm Fischer’s original observations, and another 50 for Wei and colleagues to provide further exciting insights about the regulation of cell wall ingrowth formation; however, the answer to ‘what’s next?’ is certainly expected to come much sooner. With the advent of new technologies, such as single-cell RNaseq (Ryu *et al.*, 2019), we anticipate that many new pieces of the puzzling functions and regulation of PPTC wall ingrowth will be illuminated in a much shorter period of time.

Box 1. **The PPTC: a key nexus in the sucrose transport pathway**

**Fig. 1.**
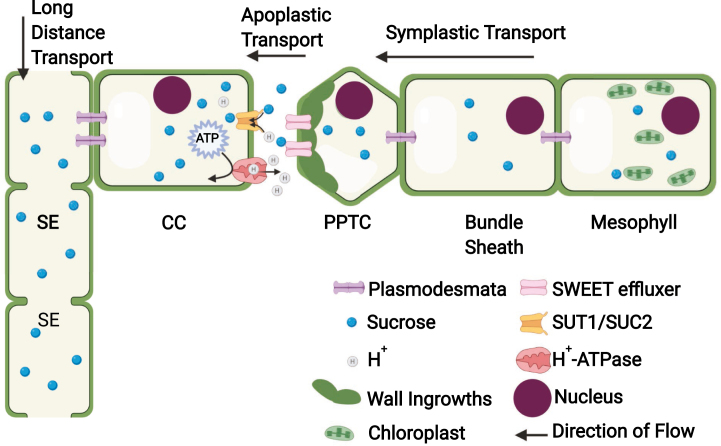
Symplastic and apoplastic transport of sucrose into the phloem.

The transport of assimilated carbon, usually in the form of sucrose, from photosynthetic tissues such as leaves to heterotrophic tissues (e.g. roots, developing leaves, and reproductive structures) is an essential process known as carbohydrate partitioning (Julius *et al.*, 2017). In many plants, including most crops species, the conduit facilitating long-distance transport, the SE–CC complex, is symplastically isolated from other cells in the vein (i.e. apoplastic phloem loading species, see Figure 1). In such plants, sucrose phloem loading is accomplished by membrane-bound H^+^/sucrose symporters of the sucrose transporter (SUT/SUC) family, which transport sucrose from the apoplast into the SE–CC (Riesmeier *et al.*, 1994; Srivastava *et al.*, 2008; Slewinski *et al.*, 2009). The Arabidopsis genome contains nine SUTs which differ in transport kinetics (high and low affinity) and specificity of expression (Kühn and Grof, 2010). In the angiosperm pan-genome, five phylogenetic clades have been described, including eudicot- and monocot-specific clades (Braun and Slewinski, 2009). Phloem loading by SUC2/SUT1 plays a key role in facilitating sucrose export from the leaf (Riesmeier *et al.*, 1994; Srivastava *et al.*, 2008; Slewinski *et al.*, 2009).

The more recent discovery of the *SWEET* family of sugar effluxers was also a major leap forward in understanding the phloem transport pathway (Chen *et al.*, 2012). Positioned at the boundary of symplastic efflux and apoplastic uptake of sucrose, these transporters appeared to be the ‘missing link’ that filled in a key gap in sucrose movement between the site of sucrose synthesis and loading into the SE–CC (Baker *et al.*, 2012). Chen and colleagues provided molecular evidence implicating PPTCs in SWEET-mediated sucrose efflux by observing SWEET11–green fluorescent protein (GFP) fluorescence signal driven by the *AtSWEET11* promoter in what the authors determined are PPTCs in sepal minor veins. The GFP accumulation resembled static puncta, which the authors suggested are caused by the characteristic cell wall ingrowths, the presence of which they confirm with electron microscopy (Chen *et al.*, 2012). Additionally, the decreased sucrose export from source leaves in *sweet11; sweet12* double mutants suggested that PPTCs performed an essential function in the sucrose transport pathway. However, the discovery of Arabidopsis *vte* mutants lacking tocopherols (vitamin E) provided unexpected confirmation—from literally the opposite direction (i.e. the cell wall interface with bundle sheath cells rather than with the SE–CC).

The Arabidopsis *vitamin e1 (vte1*) mutant, encoding a tocopherol cyclase, was identified in a screen for lipid-deficient mutants (Porfirova *et al.*, 2002). The causal gene was noted for its high similarity to the maize *Sucrose export defective1* (*Sxd1*) gene, mutants of which exhibit callose occlusion of the phloem and a corresponding reduction of sucrose export from leaves (Botha *et al.*, 2000). Because maize PPTCs lack cell wall ingrowths, Arabidopsis proved to be a better system to interrogate the essentiality of PPTC-mediated sucrose transport and its role in cell wall ingrowth formation. Maeda *et al.* (2006) later identified *vte2*, another enzyme in the tocopherol biosynthetic pathway. While both *vte1* and *vte2* mutants exhibited characteristics typical of mutants deficient in sucrose transport (i.e. starch hyperaccumulation in leaves and stunted growth), *vte2* exhibited a concomitant deposition of callose specifically between the bundle sheath and PPTC interface (Maeda *et al.*, 2006). Most significantly, the callose deposition in *vte2* was associated with an absence of normal PPTC wall ingrowth formation (Maeda *et al.*, 2006). Taken together, these results show that disruption of sucrose transport through the PPTC, either by disrupting SWEET-mediated export to the apoplastic space, or by blocking the symplastic flow of sucrose from bundle sheath cells, results in reduced sucrose export from leaves, highlighting the importance of the PPTC in the sucrose transport pathway. In addition, the results reported by Maeda *et al.* (2006) provide a direct link between sucrose accumulation in PPTCs and the formation of cell wall ingrowths.

## References

[CIT0001] Baker RF, Leach KA, Braun DM 2012 SWEET as sugar: new sucrose effluxers in plants. Molecular Plant 5, 766–768. 22815540 10.1093/mp/sss054

[CIT0002] Botha C, Cross R, Van Bel A, Peter C 2000 Phloem loading in the *sucrose-export-defective* (*SXD-1*) mutant maize is limited by callose deposition at plasmodesmata in bundle sheath–vascular parenchyma interface. Protoplasma 214, 65–72.

[CIT0003] Braun DM, Slewinski TL 2009 Genetic control of carbon partitioning in grasses: roles of sucrose transporters and *tie-dyed* loci in phloem loading. Plant Physiology 149, 71–81. 19126697 10.1104/pp.108.129049 PMC2613709

[CIT0004] Bulbert MW, Offler CE, McCurdy DW 1998 Polarized microtubule deposition coincides with wall ingrowth formation in transfer cells of *Vicia faba* L. cotyledons. Protoplasma 201, 8–16.

[CIT0005] Chen L-Q, Qu X-Q, Hou B-H, Sosso D, Osorio S, Fernie AR, Frommer WB 2012 Sucrose efflux mediated by SWEET proteins as a key step for phloem transport. Science 335, 207–211. 22157085 10.1126/science.1213351

[CIT0006] Dibley SJ, Zhou Y, Andriunas FA, Talbot MJ, Offler CE, Patrick JW, McCurdy DW 2009 Early gene expression programs accompanying trans‐differentiation of epidermal cells of *Vicia faba* cotyledons into transfer cells. New Phytologist 182, 863–877. 19383101 10.1111/j.1469-8137.2009.02822.x

[CIT0007] Edwards J, Martin AP, Andriunas F, Offler CE, Patrick JW, McCurdy DW 2010 GIGANTEA is a component of a regulatory pathway determining wall ingrowth deposition in phloem parenchyma transfer cells of *Arabidopsis thaliana*. The Plant Journal 63, 651–661. 20545890 10.1111/j.1365-313X.2010.04269.x

[CIT0008] Esau K 1967 Minor veins in Beta leaves: structure related to function. Proceedings of the American Philosophical Society 111, 219–233.

[CIT0009] Esau K, Cheadle V, Gifford E 1953 Comparative structure and possible trends of specialization of the phloem. American Journal of Botany 40, 9–19.

[CIT0010] Evert R, Eschrich W, Heyser W 1978 Leaf structure in relation to solute transport and phloem loading in *Zea mays* L. Planta 138, 279–294. 24414058 10.1007/BF00386823

[CIT0011] Fischer A 1884 Untersuchungen *ü*ber das Siebr*ö*hren-System der Cucurbitaceen: ein Beitrag zur vergleichenden Anatomie der Pflanzen. Gebrüder Borntraeger.

[CIT0012] Fowler S, Lee K, Onouchi H, Samach A, Richardson K, Morris B, Coupland G, Putterill J 1999 GIGANTEA: a circadian clock‐controlled gene that regulates photoperiodic flowering in Arabidopsis and encodes a protein with several possible membrane‐spanning domains. The EMBO Journal 18, 4679–4688. 10469647 10.1093/emboj/18.17.4679 PMC1171541

[CIT0013] Gunning B, Pate J 1969 ‘Transfer cells’ plant cells with wall ingrowths, specialized in relation to short distance transport of solutes—their occurrence, structure, and development. Protoplasma 68, 107–133.

[CIT0014] Gunning B, Pate J, Briarty L 1968 Specialized’ transfer cells’ in minor veins of leaves and their possible significance in phloem translocation. Journal of Cell Biology 37, C7. 11905215 10.1083/jcb.37.3.c7 PMC2107441

[CIT0015] Huq E, Tepperman JM, Quail PH 2000 GIGANTEA is a nuclear protein involved in phytochrome signaling in Arabidopsis. Proceedings of the National Academy of Sciences, USA 97, 9789–9794. 10.1073/pnas.170283997 PMC16943 10920210

[CIT0016] Julius BT, Leach KA, Tran TM, Mertz RA, Braun DM 2017 Sugar transporters in plants: new insights and discoveries. Plant & Cell Physiology 58, 1442–1460. 28922744 10.1093/pcp/pcx090

[CIT0017] Knoblauch M, Knoblauch J, Mullendore DL, Savage JA, Babst BA, Beecher SD, Dodgen AC, Jensen KH, Holbrook NM 2016 Testing the M*ü*nch hypothesis of long distance phloem transport in plants. eLife 5, e15341. 27253062 10.7554/eLife.15341 PMC4946904

[CIT0018] Knoblauch M, Peters WS 2017 What actually is the M*ü*nch hypothesis? A short history of assimilate transport by mass flow. Journal of Integrative Plant Biology 59, 292–310. 28276639 10.1111/jipb.12532

[CIT0019] Kühn C, Grof CP 2010 Sucrose transporters of higher plants. Current Opinion in Plant Biology 13, 287–297. 10.1016/j.pbi.2010.02.001 20303321

[CIT0020] Maeda H, Song W, Sage TL, DellaPenna D 2006 Tocopherols play a crucial role in low-temperature adaptation and phloem loading in Arabidopsis. The Plant Cell 18, 2710–2732. 17012603 10.1105/tpc.105.039404 PMC1626601

[CIT0021] Münch E 1930 Stoffbewegungen in der Pflanze [‘Movements of material in the plant’]. Jena: G. Fischer.

[CIT0022] Offler CE, McCurdy DW, Patrick JW, Talbot MJ 2003 Transfer cells: cells specialized for a special purpose. Annual Review of Plant Biology 54, 431–454. 10.1146/annurev.arplant.54.031902.134812 14502998

[CIT0023] Pate J, Gunning B 1972 Transfer cells. Annual Review of Plant Physiology 23, 173–196.

[CIT0024] Penfield S, Hall A 2009 A role for multiple circadian clock genes in the response to signals that break seed dormancy in Arabidopsis. The Plant Cell 21, 1722–1732. 19542296 10.1105/tpc.108.064022 PMC2714941

[CIT0025] Porfirova S, Bergmüller E, Tropf S, Lemke R, Dörmann P 2002 Isolation of an Arabidopsis mutant lacking vitamin E and identification of a cyclase essential for all tocopherol biosynthesis. Proceedings of the National Academy of Sciences, USA 99, 12495–12500. 10.1073/pnas.182330899 PMC129473 12213958

[CIT0026] Riesmeier JW, Willmitzer L, Frommer WB 1994 Evidence for an essential role of the sucrose transporter in phloem loading and assimilate partitioning. The EMBO Journal 13, 1–7. 8306952 10.1002/j.1460-2075.1994.tb06229.x PMC394773

[CIT0027] Ryu KH, Huang L, Kang HM, Schiefelbein J 2019 Single-cell RNA sequencing resolves molecular relationships among individual plant cells. Plant Physiology 179, 1444–1456. 30718350 10.1104/pp.18.01482 PMC6446759

[CIT0028] Slewinski TL, Meeley R, Braun DM 2009 *Sucrose transporter1* functions in phloem loading in maize leaves. Journal of Experimental Botany 60, 881–892. 19181865 10.1093/jxb/ern335 PMC2652052

[CIT0029] Srivastava AC, Ganesan S, Ismail IO, Ayre BG 2008 Functional characterization of the Arabidopsis AtSUC2 sucrose/H+ symporter by tissue-specific complementation reveals an essential role in phloem loading but not in long-distance transport. Plant Physiology 148, 200–211. 18650401 10.1104/pp.108.124776 PMC2528097

[CIT0030] Wei X, Nguyen ST, Collings DA, McCurdy DW 2020 Sucrose regulates wall ingrowth deposition in phloem parenchyma transfer cells in Arabidopsis via affecting phloem loading activity. Journal of Experimental Botany 71, XXXX–XXXX. 10.1093/jxb/eraa246 32433727

[CIT0031] Wimmers LE, Turgeon R 1991 Transfer cells and solute uptake in minor veins of *Pisum sativum* leaves. Planta 186, 2–12. 24186568 10.1007/BF00201491

[CIT0032] Zhou Y, Andriunas F, Offler CE, McCurdy DW, Patrick JW 2010 An epidermal‐specific ethylene signal cascade regulates trans‐differentiation of transfer cells in *Vicia faba* cotyledons. New Phytologist 185, 931–943. 20085619 10.1111/j.1469-8137.2009.03136.x

